# Enhancement of Drought Tolerance in Cucumber Plants by Natural Carbon Materials

**DOI:** 10.3390/plants8110446

**Published:** 2019-10-24

**Authors:** Tae Yoon Kim, Sang-Hyo Lee, Hara Ku, Seung-Yop Lee

**Affiliations:** 1 Department of Biomedical Engineering, Sogang University, Baekbeom-ro 35, Mapo-gu, Seoul 04107, Korea; kimtaeyoon@sogang.ac.kr (T.Y.K.); bpdl1@sogang.ac.kr (S.-H.L.); haraku617@sogang.ac.kr (H.K.); 2 Department of Mechanical Engineering, Sogang University, Baekbeom-ro 35, Mapo-gu, Seoul 04107, Korea

**Keywords:** drought, cucumber, abiotic stress, carbon nanotube, shungite, water stress

## Abstract

Stress induced by climate change is a widespread and global phenomenon. Unexpected drought stress has a substantial effect on the growth and productivity of valuable crops. The effects of carbon materials on living organisms in response to abiotic stresses remain poorly understood. In this study, we proposed a new method for enhancing drought tolerance in cucumber (*Cucumis sativus* L.) using carbon nanotubes and natural carbon materials called shungite, which can be easily mixed into any soil. We analyzed the phenotype and physiological changes in cucumber plants grown under conditions of drought stress. Shungite-treated cucumber plants were healthier, with dark green leaves, than control plants when watering was withheld for 21 days. Furthermore, compared with the control cucumber group, in the shungite-treated plants, the monodehydroascorbate content of the leaf, which is a representative marker of oxidative damage, was 66% lower. In addition, major scavenger units of reactive oxygen species and related drought stress marker genes were significantly upregulated. These results indicate that successive pretreatment of soil with low-cost natural carbon material improved the tolerance of cucumber plants to drought stress.

## 1. Introduction

There are predictions that a significant increase in agricultural production (e.g., food, feed, fibers, and bio-energy) will be needed in the next 50 years to meet global food demands for the growing human population, which is estimated to reach approximately 9.7 billion [1,2,3]. This challenge is further exacerbated by the projected climate change scenario including water shortage [4]. In particular, all low- and mid-latitude countries, including China, Israel, and those in Europe, Central Asia, and the southwestern United States are predicted to experience severe weather in addition to global warming, water deficiency, and high temperatures. A recent study analyzed data from reports published from 1980 to 2015 and found that up to 21% and 40% yield reductions in wheat (*Triticum aestivum* L.) and maize (*Zea mays* L.), respectively, occurred mainly due to drought on a global scale [5]. Water shortage causes a substantial decline in crop yields due to the negative effects on plant growth through physical damage, physiological disruption, biochemical change, and reproduction problems [6,7]. Therefore, the development of new technology is urgently needed to maintain crop yields under severe water-deficit stress. 

Currently, there are several ways to overcome these difficulties. Development of smart farms, desalination of seawater, and development of genetically modified (GM) crops through genetic engineering are some of these ways. However, most of these technologies are costly and have numerous limitations, including food safety issues. Therefore, the development of new technology is required to meet the food demands of an ever-increasing global population, particularly under the challenges posed by climate change. 

In this study, we tested a new method for enhancing drought tolerance in plants using industrial or natural products that can be easily mixed into any soil. Among various candidate materials, several studies have shown that substances with a high content of carbonaceous materials of various structures have a positive or negative effect on plant growth [8,9,10]. Mixing single- or multiwalled carbon nanotubes (SWCNT or MWCNT, respectively) with the soil enhanced growth by 1.5- or 2-fold, respectively. The treatment of the soil with MWCNT was shown to induce physiological changes in tomato (*Solanum lycopersicum* L.) and upregulate the expression of multiple stress-response genes [11]. 

Shungite, a natural carbon material, is a mineral mined mainly in Russia. Its natural products have been used for preparing eco-friendly construction materials and water purification filters for over 100 years. It is composed of 30%–98% carbon and has a fullerene-like regular structure at a ratio of 0.001%–0.0001% [12]. Until recently, published studies on the response to abiotic stress following the application of these carbon materials have been rare. We, therefore, proposed a novel method of improving drought tolerance, based on the application of MWCNT and shungite.

Several physiological evaluation criteria were established to verify the effectiveness of these applications based on published reports. It is essential to understand the physiological, biochemical, and ecological interventions relating to drought stress for improved crop management. Unfavorable environmental conditions inflict damage on stressed plants mainly through the generation of oxidative and osmotic stresses [13]. Oxidative stress results from the generation of reactive oxygen species (ROS), such as superoxide (O_2_^−^) and hydroxyl (OH^−^) ions, hydrogen peroxide (H_2_O_2_), and singlet oxygen. Although ROS are mainly produced in plants by organelles involved in energy transformation, they are also generated at other sites in plant cells, such as peroxisomes, apoplasts, endoplasmic reticulum, and the cytosol [14,15].

Under normal growth conditions, plants produce ROS and their accumulation is balanced by the action of different antioxidants in the cell [16]. In plants, there are enzymatic and nonenzymatic antioxidants. The first line of defense against ROS accumulation is superoxide dismutase (SOD), which dismutates the O_2_- radical to H_2_O_2_ [15,17]. Catalase (CAT) and peroxidase (POD) are two other enzymes that scavenge H_2_O_2_ and prevent its accumulation to toxic levels. These two enzymes have been shown to have different affinities for H_2_O_2_. In particular, CAT has low affinity for H_2_O_2_, and may be responsible for scavenging most of the H_2_O_2_ [15,17]. However, under persistently stressful conditions, ROS are produced at a high rate and are not scavenged adequately by the different antioxidants and, thus, accumulate in the plant cell. ROS accumulation is harmful to the different macromolecules within the cell, as they cause degradation of proteins, DNA, and lipids, in addition to increasing membrane permeability [17,18].

Most studies on oxidative stress and the activities of antioxidant enzymes in response to drought stress have yielded inconsistent results. This is because ROS accumulation and the upregulation of antioxidant enzymes are dependent on plant species, plant genotype, stress severity, stress duration, plant development, and metabolism [18,19].

Other major physiological responses include activation of the abscisic acid (ABA) signaling pathway and subsequent gene expression patterns in living organs under stressful conditions [20]. Cellular water-deficit stress triggers marked changes in gene expression, which can be used to define the response of a plant to an environmental condition [21]. Among these responses is the regulation of CO_2_ uptake and transpiration in plants by the stomata. Genetic manipulation of stomata development and movement has been shown to alter water-use efficiency (WUE), mainly by regulating transpiration, which eventually affects drought tolerance [22,23]. Stomatal movement is regulated by many environmental factors as well as phytohormones [24,25]. Among these, the phytohormone ABA mediates the early response to soil drying by limiting the stomatal aperture to prevent water loss [26,27,28]. In recent years, significant progress has been made in the understanding of the molecular mechanisms of ABA perception and signal transduction in *Arabidopsis thaliana* [29,30]. Initially, ABA is detected by the receptor proteins pyrabactin resistance 1/PYR1-like/regulatory components of ABA receptors (PYR1/PYL/RCAR). In the presence of ABA, these receptors interact with type 2C protein phosphatase (PP2C), thereby negatively regulating ABA signaling and inhibiting the phosphatase activity of PP2C. Subsequently, SNF1-related protein kinase 2s (SnRK2s) are activated by PP2Cs and phosphorylated leucine-zipper ABA-responsive element (ABRE)-binding proteins/ABRE-binding factors (AREBs/ABFs), which bind directly to the ABREs of stress-responsive genes, to stimulate the relevant transcription factors [31,32].

Cucumber (*Cucumis sativus* L.) is one of the top 10 vegetable crops worldwide. Cucumber and other Cucurbitaceae plants have a high transpiration rate and sensitivity to drought [22]. Therefore, in the development of a technology to induce drought resistance, verification in cucumber provides reasonable insight into extending the application of the technology to most other crops. In this study, we investigated a novel approach to manage water-deficient conditions using unique inorganic substances and mechanically engineered products. To identify the physiological differences between the control and shungite-containing soil groups, the following characteristics were examined: morphology; phenotype; enzymatic activities of SOD, CAT, and POD; rate of stomatal closure under drought stress; ABA sensitivity; and relative expression of ABA-signaling genes. 

## 2. Results

### 2.1. Carbon Effect under Drought Stress

**Figure 1:**
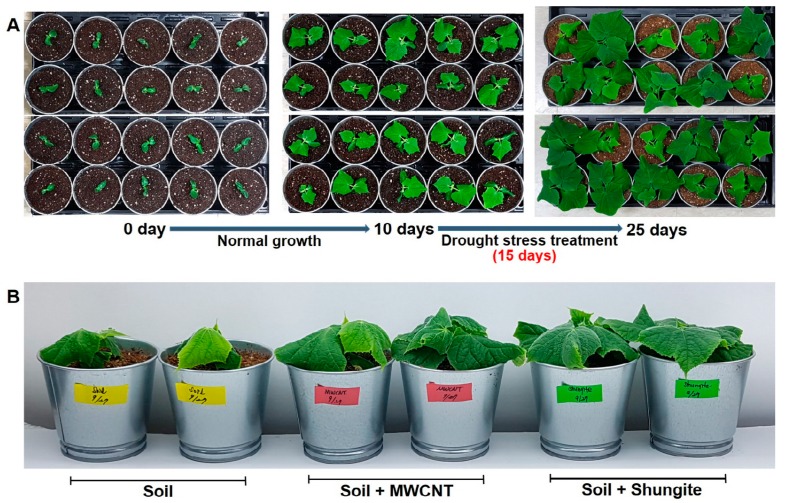
Improving wilt symptoms in leaves through the addition of MWCNT and shungite, under drought conditions. (**A**) Overview of the experimental design. (**B**) Plant status at the time of sampling. Ten-day-old cucumber (“Eun-sung”) plants were moved to drought conditions. Plants were grown for 15 days under drought conditions. MWCNT: multiwalled carbon nanotubes.

As a well-known carbon material, MWCNT was selected and compared with shungite. Recent studies have reported that MWCNT has a positive effect under drought stress at different growth stages and plants [33,34,35]. Verification of drought stress was performed by mixing two carbon materials (MWCNT and shungite, 0.067%) with normal soil. For 10 days under normal growing, no water was added to plants following the four-leaf stage (Figure 1A). After 15 days, wilting and yellowing plants were observed in only the normal soil group. Conversely, cucumbers grown in both carbon-containing soils showed a healthy phenotype. In particular, the best growth was observed in the shungite-treated group (Figure 1B). Plants grown in soil treated with shungite were the most tolerant to drought. Therefore, all subsequent experiments were carried out using only shungite as an additive material.

### 2.2. Enhanced Drought Tolerance of Shungite-Treated Plants 

Plant growth is mainly accomplished by cell division, enlargement, and differentiation. Drought impairs mitosis and cell elongation, resulting in reduced plant height, leaf size, and stem growth [36]. Limited cell growth is mainly due to the loss of turgor pressure and poor water flow from the xylem to the neighboring cells [37]. Reduced turgor pressure and a slow rate of photosynthesis under drought conditions limit leaf expansion. Plant height, leaf size, and stem growth are significantly reduced under limited water conditions.

**Figure 2:**
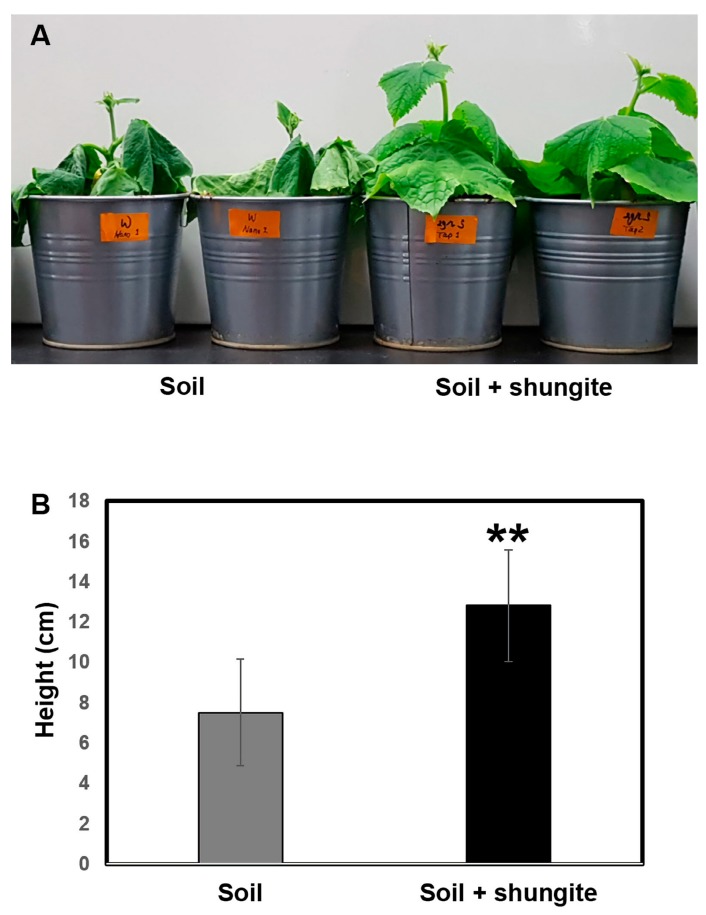
Effects of shungite as a physiological indicator of drought tolerance in cucumber. (**A**) Shungite-added group shows tolerance under drought conditions. Representative image after 21 days. (**B**) Comparison of plant heights. Error bars represent mean ± standard deviation (SD), (*n* = 8). Asterisks indicate statistical significance (Student’s *t*-test, ** *p* ≤ 0.05).

Plants were treated with distilled water at 2 day intervals for a total of 12 days. After withholding watering for 21 days, the leaves of cucumber plants in shungite-containing soil displayed darker green color, milder wilt symptoms, and sustained turgor pressure (Figure 2A). In contrast, cucumber plants in normal soil demonstrated drought stress symptoms (cucumber leaves no longer rehydrated at night or in the early morning) after watering was withheld for 21 days. The control group presented a smaller phenotype under drought stress, whereas plants in the shungite treatment group were twice the height of the control plants, suggesting a maintained tolerance phenotype (Figure 2B).

### 2.3. Effects of Shungite on Drought Tolerance

Drought stress leads to membrane peroxidation that derives from cell wall degradation and membrane damage. The production of ROS is strongly influenced by stress factor responses in plants [38]. Malonyldialdehyde (MDA) is well known as a marker of oxidative damage to cells [39]. 

**Figure 3:**
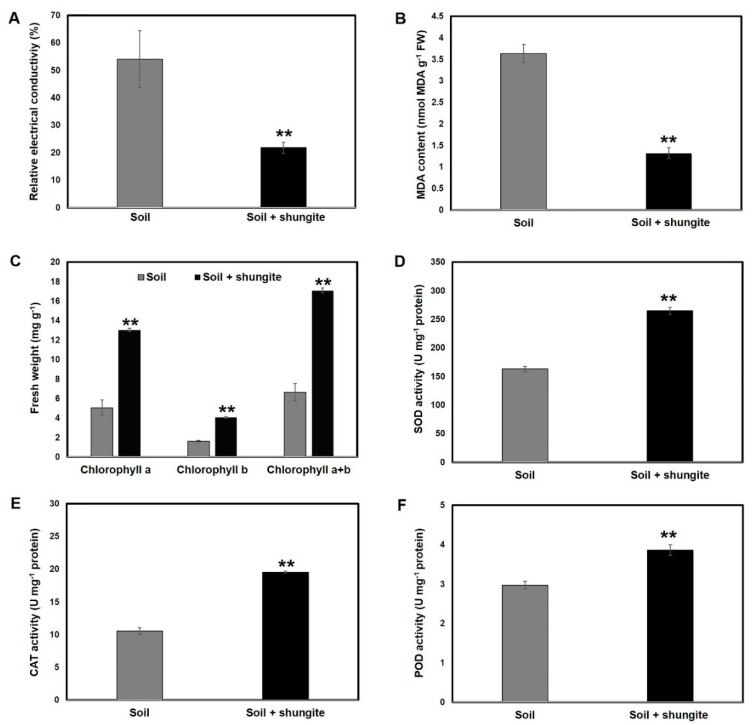
Effects of shungite on physiological indicators of drought tolerance in cucumber. (**A**) Determination of relative electrical conductivity. (**B**) Comparative analysis of MDA accumulation in cucumber leaves. (**C**) Determination of total chlorophyll content in the same leaf area (3 cm^2^). (**D**–**F**) Comparison of ROS scavenger activity under drought stress. The leaf SOD, POD, and CAT activities in cucumber plants. Experiments were performed using three randomly collected leaves from 18 plants on day 21, at which point severely wilted plants were observed in the control group. All experiments were performed in triplicate, and the average is represented in graph. Error bars represent the mean ± SD. Asterisks indicate statistical significance (Student’s *t*-test, ** *p* ≤ 0.05). MDA: Malonyldialdehyde; ROS: reactive oxygen species; SOD: superoxide dismutase; POD: peroxidase; CAT: Catalase.

In order to examine the effect of shungite on cucumber plants exposed to water-deficit stress, the leaf electrical conductivity (EC), leaf MDA, and chlorophyll content of leaves were determined. Based on the EC measurements and examination of damage to intracorporeal cells, the shungite-treatment group had about 65.7% lower value than did the control group (Figure 3A). The leaf MDA of the plants in the soil containing shungite was about 1.3 nmol/g, which was 66% lower than that of the control plants (Figure 3B). In addition, the amount of total chlorophyll in a 3 cm^2^ area was 2.7 times higher than that in the control group (Figure 3C). Taken together, these results indicated that the shungite-treated group was less affected than the control group when exposed to drought stress.

ROS levels are kept in check by scavengers, including SOD, CAT, and POD. However, excessive stress impairs homeostasis and induces various damage, such as cell wall degradation, membrane damage, and DNA and protein degradation [40].

We investigated the effect of shungite on the corresponding enzymatic system in cucumber plants in response to drought stress by determining the activity of SOD, CAT, and POD in leaves. As shown in Figure 3D, shungite addition resulted in a substantial increase in leaf SOD activity, which peaked at 150 U/mg when compared with 70 U/mg in the control. This indicated that shungite significantly increased SOD activity in the leaves of cucumber plants under drought stress. Moreover, in shungite-treated cucumber plants, leaf CAT and POD activity was 2.7 and 1.6 times than that in the control plants (Figure 3E,F).

### 2.4. Changes in Stomatal Movement and Development 

**Figure 4:**
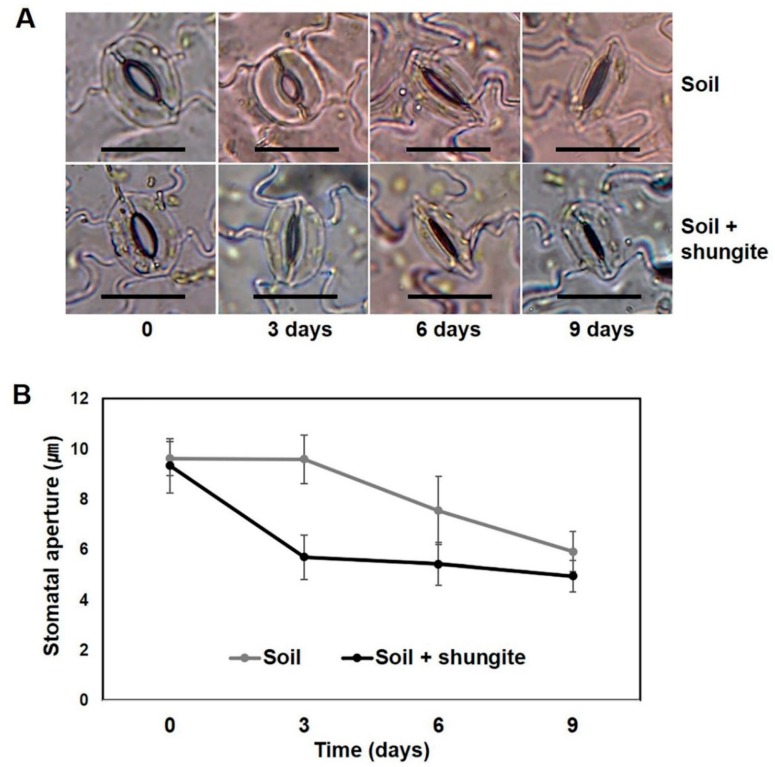
Time-course changes in stomatal aperture on cucumber leaves’ response to shungite under drought stress. (**A**) Stomatal aperture in detached abaxial epidermal strips under drought stress. Scale bar = 20 µm. (**B**) Data are represented as the mean of 15 randomly selected stomata from three biological replicates (±SD). Different letters indicate significant differences (Student’s *t*-test, *p* ≤ 0.05).

Next, we investigated how the changes in transpiration were associated with stomatal movement in the plants grown in the shungite-containing soil. Under well-watered conditions, there were no differences in stomatal aperture between the normal plants and the shungite-treated plants (Figure 4). Drought induced marked decreases in the stomatal aperture in plants of both groups. Although drought-induced stomatal closure was more significant after 3 and 6 days, no significant differences were found after 9 days, and the stomatal aperture was significantly smaller in shungite-treated plants than in control plants after 3 and 6 days (Figure 4B).

### 2.5. Sensitivity of the Stomatal Aperture to ABA 

**Figure 5:**
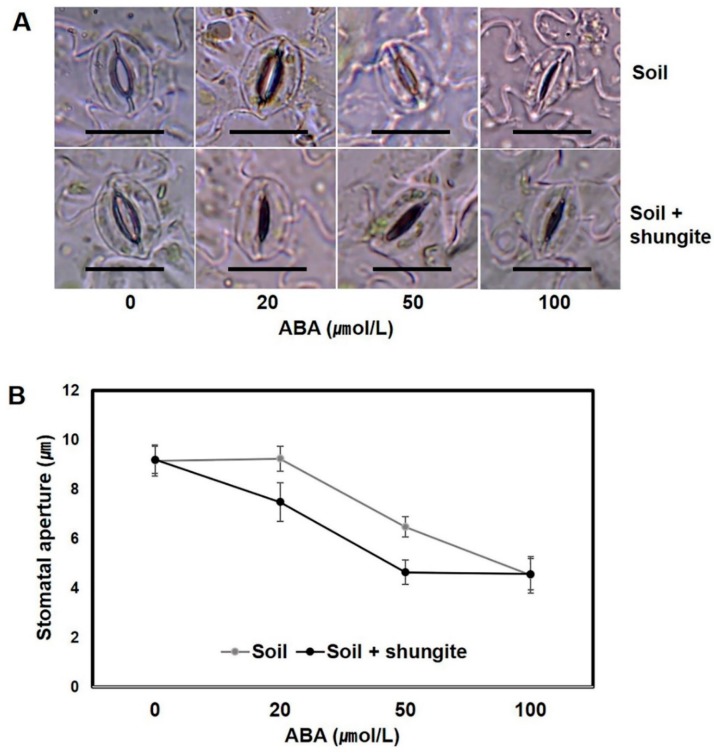
Effect of shungite on the sensitivity of the stomatal aperture to ABA. (**A**) The effect of ABA concentration on stomatal aperture in detached abaxial epidermal strips. Scale bar = 20 µm. (**B**) Data are represented as the mean of 15 randomly selected stomata from three biological replicates (±SD). Different letters indicate significant differences (Student’s *t*-test, *p* ≤ 0.05). ABA: abscisic acid.

To investigate the relationship between shungite-induced stomatal movement and ABA sensitivity, we compared water loss from detached leaves grown in normal soil and shungite-containing soil. Stomatal closure was induced by exogenous ABA at concentrations as low as 20 μM in plants grown in shungite soil, whereas 50 μM ABA was needed for plants grown in normal soil (Figure 5). These results suggest that the stomata in plants grown in shungite soil had increased sensitivity to ABA when compared with plants grown in normal soil.

### 2.6. Mechanisms of Stomatal Closure under Drought Stress

An important defense response of plants to drought is the closure of the stomatal guard cells to minimize water loss [41,42,43,44]. The opening and closing of guard cells are regulated by turgor pressure, which is further regulated by the major plant hormone ABA. As ABA levels increase because of external stress, expression of members of the sensor protein families, such as PYR/PYL/RCAR, is upregulated. Subsequently, it induces reactions in the next cascade, including defense responses and lowering of the turgor pressure by releasing water, potassium, and chloride ions. In response to drought stress, plants modulate ABA levels in stomatal cells by binding to sensor proteins and triggering the phosphorylation of downstream proteins, such as those in the SnRK2 family. Finally, the stomatal guard cells close, and various stress response genes are activated.

### 2.7. Relative Expression Patterns of ABA-Signaling Genes 

**Figure 6:**
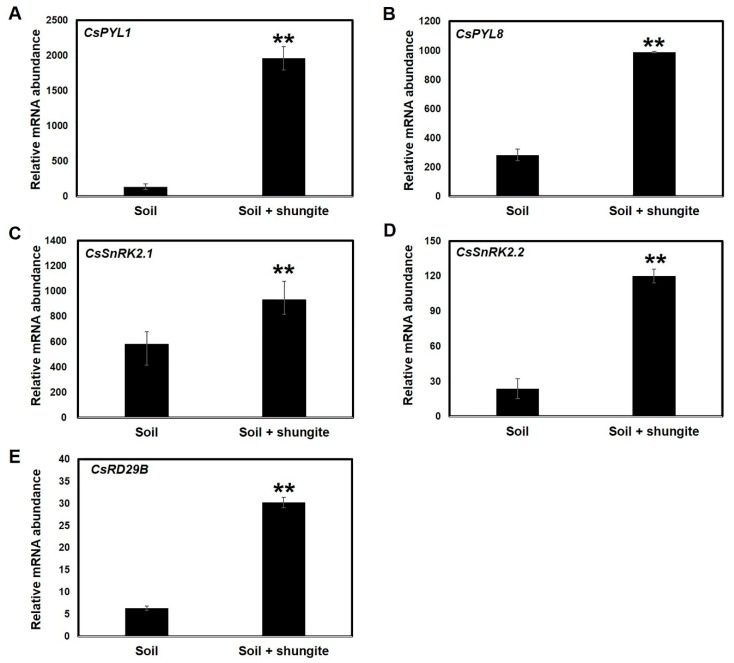
Expression profiling of cucumber ABA signaling genes in response to drought stress (**A**–**E**). Relative expression of ABA signaling transcripts was determined by real-time PCR 21 days after drought treatment. The Y-axis represents relative expression levels normalized by the elongation factor 1-aplha (*CsEF1α*) gene (*Csa006172*). Three independent experiments with three biological replicates were averaged (± SD). Statistically significant differences between control and shungite treatment groups at corresponding time points are indicated by asterisks (Student’s *t*-test, ** *p* ≤ 0.05).

To better understand the molecular changes induced by shungite material on ABA signaling in the cucumber plants, we analyzed gene expression pattern after exposure to water-deficit stress by selecting *CsPYL1*, *CsPYL8*, *CsSnRK2.1,* and *CsSnRK2.2* as marker genes associated with sensor proteins and downstream *CsRD29A* genes. Upon drought stress (21 continuous days), sensor and defense-response genes were consistently expressed at high levels in the plants grown in the shungite soil. Compared with the normal growth conditions, drought stress-induced expression of *CsPYL1*, *CsPYL8*, *CsSnRK2.1*, *CsSnRK2.2,* and *CsRD29A* increased 14.2-, 3.5-, 1.6-, 5-, and 4.7-fold, respectively (Figure 6). 

## 3. Discussion

Several studies have shown that substances with a high content of carbonaceous materials and MWCNT have a positive effect on plant growth [9,10,11]. Shungite is a natural element containing high compositions of carbon. Under drought stress, the shungite treatment group showed a more tolerant phenotype compared with the MWCNT group (Figure 1). The phenotype difference could be due to the discrepancy in physical properties between carbon nanotubes and natural shungite, such as pore size and the degree of different combinations. 

When the main phenotype was analyzed, the true leaves of cucumber grown in normal soil showed chlorophyll degradation and a yellowing and drying crispy phenotype after 21 days of drought; however, the plants grown in shungite-containing soil remained healthy and presented large leaves (Figure 2). These results indicate that the water pressure inside the plant was maintained and that water lost due to drought stress was relatively low. Leaf growth is a flexible process during which the final shape and size of the organ is adjusted to the environment. During development under abiotic stress, cell expansion in leaves is partly controlled through the regulation of apoplastic ROS homeostasis [45]. Based on these phenotypes and recent backgrounds, we analyzed the physiological differences between the two groups. 

The degrees of leaf cell damage were compared by estimating EC and total chlorophyll content. We found that the experimental group with added shungite suffered the least damage when exposed to drought (Figure 3). ROS typically damages the cells in tissues, and this destroys the plant or impairs animal homeostasis and increases cell membrane permeability [45,46]. Therefore, MDA accumulation is used widely as a marker of oxidative stress in animals and plants [47,48]. MDA levels in the plants in the shungite group were low, about one third of that in the control group, indicating that the plants experienced lower levels of oxidative stress (Figure 3).

Reduced oxidative stress in vivo indicates that ROS are cleared through homeostasis. Hyper-sensitive phenotypes under drought stress condition have been reported in a catalase-deletion mutant in *Arabidopsis* and other plants, [30,49]. This might be a key factor in conferring resistance to drought stress in plants. Therefore, we measured the activities of CAT, SOD, and POD, which are representative ROS scavengers related to drought stress [14,50]. As a result, the activity of these three scavengers was 1.2–2-fold higher compared with those in the control group (Figure 3D–F). The findings of our study indicate that the activity of these enzymes is high in plants grown in the shungite-treated soil. Accordingly, the lower accumulation of ROS in the plants grown in the shungite-treated soil compared with the control plants, may be attributed to increased ROS scavenging. The high activity of scavengers reduces damage at the cellular level and eventually plays an important role in presenting a health phenotype.

Another plant defense response involves minimizing water loss through the closure of the stomata. We observed the stomatal guard cells closing at 3 day intervals under drought stress. As a result, it was confirmed that the stomatal guard cells in plants grown in the shungite group closed quickly (Figure 4). These results are consistent with the findings of recent studies that demonstrated drought resistance by grafting cucumber root to other species [22]. Stomatal opening and closing is controlled by turgor pressure, which is, in turn, controlled physiologically by ABA [28,41,42]. Hypersensitivity to ABA in plants is associated with enhanced tolerance to drought via expression of response genes and regulation of stomata closure [22]. This study shows the increased sensitivity of stomatal closing movement to drought in the leaves of plants grown in the shungite-treated soil, as shown in the low quantity of water loss from intact leaves (Figure 5). ABA signaling is mediated by three classes of proteins: PYR1/PYL/RCAR, PP2C, and SnRK2. Increased ABA accumulation was correlated with the transcription of the ABA receptor genes *PYL1* and *PYL8*. Additionally, *Arabidopsis pyl1-* and *pyl8*-deletion mutants confer an ABA-insensitive phenotype and ABA-induced stomatal closing [51].

Based on the relative expression levels of the transcripts of the ABA receptor or transporter genes (*PYL1*, *PYL8*, *SnRK2.1,* and *SnRK2.2*), the stress response genes were upregulated in response to water deficit in plants grown with shungite (Figure 6). *Arabidopsis* homologous *RD29A* and *RD29B* genes are representative marker gene for drought tolerance positioned downstream of the signal transduction pathway [52]. The drought-induced activities of the *AtRD29A* and *AtRD29B* promoters were monitored in soybean via fusions with the visual marker gene β-glucuronidase (*GUS*). Both *AtRD29A* and *AtRD29B* promoters were significantly activated in soybean plants subjected to over 9 days of dry-down conditions [53]. The expression of *RD29B* genes is regulated by various transcription factors, such as those in the *MAPK* kinase family, *WRKY* gene family, and *PYL-SnRKs* signaling cascades. Transgenic *Arabidopsis* plants overexpressing *TaWRKY2* exhibited increased salt and drought tolerance compared with the controls. Overexpression of *TaWRKY19* conferred tolerance to salt, drought, and freezing stress in transgenic plants. Both *TaWRKY2* and *TaWRKY19* enhanced the expression of several marker genes, including *AtRD29B*, and are bound to the promoters, thereby conferring a drought-tolerant phenotype to *Arabidopsis* transgenic lines [54,55]. In these cases, the *CsRD2B* gene was markedly upregulated under drought stress (Figure 6E), which was consistent with findings from previous studies in plants [55].

**Figure 7:**
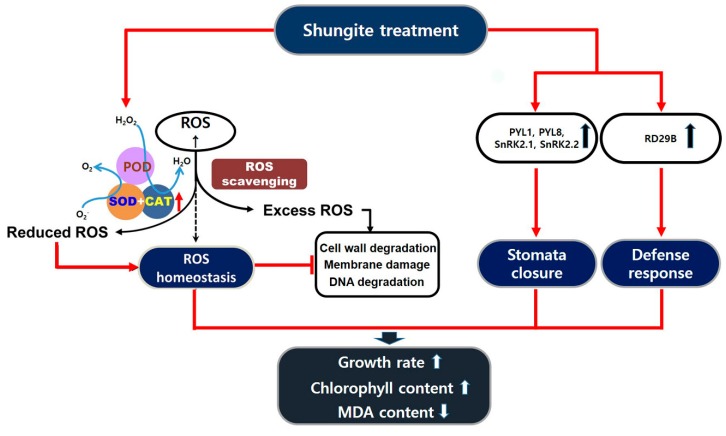
Schematic diagram of shungite induced tolerance in cucumber under drought stress. Red solid arrows indicate plant affected by shungite directly. Drought stress induces ROS production and various damages, including cell wall degradation, membrane damage, and DNA and protein degradation. The application of shungite to soil increases the activity of ROS scavengers and reduces cellular damage. This leads to better growth rate and higher chlorophyll content. It also triggers the expression of genes for stomata closure and defense reaction.

In conclusion, drought stress severely impacts plant development, growth, and fertility. Abscisic acid (ABA)-mediated closure of stomata is one of the fastest processes induced by drought [56]. Prolonged drought stress and increased stress intensity result in further acclimation reactions, including cell wall modifications and activation of the antioxidant system [57,58]. Under severe drought stress, shungite leads to stomata closing quickly and increases to the activity of antioxidants. As a result, the cell wall is less damaged and the growth inhibition effect is also reduced, as illustrated in the schematic diagram (Figure 7). This study confirmed that a low-cost natural carbon material may increase the drought tolerance of plants. Although further studies are needed to elucidate the mechanisms of stress tolerance in other plants, these findings may propose an innovative solution for major agricultural challenges induced by global warming and water scarcity. 

## 4. Materials and Methods 

### 4.1. Cucumber Plant Growth for Experiments

Peeled cucumber seeds of “Eun Sung” (Heung-nong Co., South Korea) were surface-sterilized for 1 min in 70% ethanol, and then for 15 min in 2% hypochlorite solution. After three washes, the seeds were incubated for 3 days in basal Murasigae & Skoog (MS) medium (Duchefa) containing 3% sucrose, 500 mg/L 2–(N–morpholino)ethanesulfonic acid (MES), and 0.8% phyto agar, at pH 5.8, and 23 ± 1 °C in the dark. The plates were transferred to long-day light conditions and allowed to grow for a further 4 days. Whole seedlings were transferred to normal soil (Sunshine^®^ Professional growing Mix, Sun Gro Horticulture).

### 4.2. Drought Stress Treatments

#### 4.2.1. Carbon Effect under Drought Stress

Prepared cucumber seedlings were transplanted in iron pots (upper diameter 10.5 cm; height 9.5 cm) filled with soil or soil including 0.067% carbon material, such as MWCNTs (carbon content >97%, outside diameter <7 nm, inside diameter: 2–5 nm, length: 10–30 μm, product cord: KRU4300-3, KOREA NANOMATERIALS Co, USA) and shungite powder (300 mesh, outside diameter < 2mm, Karelia, Russia). The 0.067% carbon contents have shown the strongest phenotype in our previous plant growth test (data not shown). The seedling in the pot was irrigated with 100 mL of distilled water at 2 day intervals for a total of 10 days to maintain an optimal moisture level. For drought stress, watering was withheld for 15 days. All growth conditions in a growth chamber were fixed at 24 ± 1 °C with 600 μmol photons m^−2^ s^−1^ of light supplied for 16 h during the day. 

#### 4.2.2. Evaluation of Shungite-Treated Plants under Drought Stress 

Cucumber seedlings were transferred to iron pots (upper diameter 12 cm; height 14 cm) filled with normal and shungite-added soil (0.067% carbon content, 2g(v)/L(v)). For fifteen days, the seedlings were treated at 2 day intervals with distilled water, with 18 seedlings per group. After that, watering was withheld for 21 days. The heights were measured by randomly selecting 10 individuals from each group, excepting the smallest and the largest, and averaging eight plants. 

### 4.3. Determination of MDA Content, Relative Electrical Conductivity, and Chlorophyll Contents

The MDA content was determined in leaves at 21 days under drought stress, based on the previous method [50]. Three leaves were randomly collected from three of the 18 plants; 0.3 g fresh weight (FW) sample was homogenized in 5 mL of 5% trichloroacetic acid (TCA). The homogenate was centrifuged for 15 min at 8000 × *g*. The supernatant from each sample was blended with 2.5 mL of thiobarbituric acid (TBA), and then the mixture was heated at 100 °C in a water bath for 20 min and immediately cooled on ice. After that, the mixture was centrifuged at 10,000 × *g* for 5 min, and the absorbance of the associated supernatant was measured at 532 and 600 nm. The MDA content in cucumber leaves was obtained based on its molar extinction coefficient (155 mM^−1^ cm^−1^) and denoted as μmol MDA g^−1^FW.

The relative electrical conductivity in cucumber leaves was checked at 21 days, using the process described by Yang et al. [59]. Again, three leaves were collected per treatment, and then, each 0.1 g FW sample was minced, placed in a 50 mL Falcon tube, and mixed with 10 mL distilled water. After the mixture was incubated in a calorstat set at 32 °C for 2 h, the initial electrical conductivity (S1) was measured. After that, the mixture was boiled to 100 °C for 30 min and then cooled to room temperature to measure its final electric conductivity (S2). Distilled water was used as the blank control, with electrical conductance values of zero (S0). The relative electrical conductivity (REC) was determined using the formula (1):

REC = (S1-S0)/(S2-S0) × 100(1)

The contents of leaf chlorophylls were determined at 21 days, using the method by Ashraf [60]. Three leaves were randomly collected from three different plants in each group. Samples of 0.3 g fresh-eight were cut into 3 cm × 3 cm segments and extracted overnight with 100% ethanol at 4 °C. The mixture was centrifuged at 13,000 rpm for 5 min, and the supernatant was collected for the measurement at 645 and 663 nm using a spectrophotometer. Chlorophyll concentration was estimated following the equations from Arnon [61], as follows (2)–(4):

Chlorophyll a (µg/mL) = 12.7 × A663 − 2.69 × A645(2)

Chlorophyll b (µg/mL) = 22.9 × A645 − 4.68 × A663(3)

Total chlorophyll (µg/mL) = 20.2 × A645 + 8.02 × A663(4)

### 4.4. Enzyme assays

The activity of three antioxidant enzymes (SOD, POD, and CAT) in cucumber tissues was measured using commercial assay kits. Three leaves were randomly collected from three of the 18 plants; harvesting samples were frozen at –20 °C and ground in the presence of dry ice after harvesting. Each ground sample was transferred to a 1.5 mL tube, following which, 1 mL of 50 mM ice-cold phosphate buffer (pH 7.8) containing 1 mM ethylene diamine tetra acetic acid (EDTA) was added. The samples were vortexed for 5 min and incubated for 1 h in ice. The homogenate was centrifuged at 15,000 × *g* for 15 min at 4 °C. Enzyme assays were performed using the supernatant.

The SOD activity of cucumber samples was measured using a SOD Assay Kit-WST (Dojindo, Japan). The kit works on the principle that the superoxide anion reduces WST ((2-(4-iodophenyl)-3-(4-nitrophenyl)-5-(2,4-disulphophenyl)-2H-tetrazolium, mono sodium salt), producing yellow formazan, which can be measured using a spectrophotometer at 450 nm. Antioxidants within the samples inhibit yellow WST formation. Briefly, cucumber extract was mixed with WST solution. The samples were then treated with an enzyme solution and incubated at 37 °C for 20 min. Absorbance was measured at 450 nm using a spectrophotometer. The inhibition rate of formazan formation was calculated as (5):

[(Ablank 1 − Ablank 2) − (Asample − Ablank 2)]/(Ablank 1 − Ablank 3) × 100(5)

The rates of inhibition were then converted to SOD activity for a specific concentration of protein. All experiments were repeated three times.

The CAT activity of cucumber samples was measured using a CAT Assay Kit (Biomax, Korea). Catalase reduces H_2_O_2_ into water and oxygen. When H_2_O_2_ reacts with the probe reagent and horseradish peroxidase in the kit, red resorufin is formed. The amount of reduced resorufin corresponds to the amount of antioxidant activity within the sample. The absorbance of resorufin was measured using a spectrophotometer at 560 nm. All experiments were repeated three times. The POD activity of cucumber samples was measured using a POD Assay Kit (Biomax, Korea). POD was measured using a probe and horseradish peroxidase. The reduced final reaction product, resorufin, was measured at 560 nm using a spectrophotometer.

### 4.5. Stomatal Bioassay and ABA Sensitivity Experiment

Stomatal bioassays were performed using intact leaves (four-week-old plants) from control soil and shungite-treated soil. Epidermal strips from fully expanded intact leaves were peeled from the abaxial surface with forceps, and mesophyll cells were removed from the epidermis [22]. The time course of drought stress-induced changes in stomatal aperture were observed at 3 day interval and examined under a microscope. In ABA sensitivity assays, using four-week-old plants, stomatal opening was promoted by incubating the epidermis in stomatal opening buffer (10 mM KCl, pH 6.2, adjusted with KOH) for 2 h for equilibration and stomatal opening in light 100 μmol/m^2^s. Additional incubation for 30 min was done in stomatal opening buffer supplemented with 0, 20, 50, and 100 μM ABA (Sigma–Aldrich, USA). All experiments were demonstrated as the mean of 15 randomly selected stomata from three biological replicates. 

### 4.6. RNA Extraction and Real-Time PCR Analysis

Upon harvesting, the cucumber leaves were frozen in liquid nitrogen immediately, and stored at −80 °C until use for RNA extraction. Total RNA was extracted from cucumber leaves using the RNeasy kit (QIAGEN, Hilden, Germany), according to the manufacturer’s instructions. DNA contamination was removed with a purifying column. About 0.2 μg of total RNA was used for reverse transcription, and a quantitative real-time PCR (qRT-PCR) assay was performed using a QuantiTech SYBR Green RT-PCR kit (QIAGEN, Hilden, Germany) and LightCycler96 system (Roche Life Science). Each reaction (10.1 μL) consisted of 5 μL Master Mix. Reaction conditions were as follows: denaturation at 95 °C for 15 min, followed by 40 cycles of denaturation at 95 °C for 20 s, annealing at 58 °C for 20 s, and extension at 72 °C for 20 s. The transcript levels of the *CsPYL1, CsPYL8, CsSnRK2.1, CsSnRK2.2, CsRD29B* genes were normalized by the transcript level of *CsEF1α* as a reference gene, and mRNA was quantified using LC96 software version 1.1.0.1320. Experiments were carried out using three independent biological samples, and each qRT-PCR reaction included three technical replications. Gene and primer sequences were obtained based on the previous study, with minor modifications [22]. The sequences of primers are given in the supporting information (Table S1).

### 4.7. Statistical Analysis

All the experiments were arranged in a completely randomized design and performed in triplicate, and the mean is represented in the graphs. For stomatal aperture analysis, each replicate is the average aperture of 15 randomly selected stomata from three biological replicates from among 12 plants under each treatment. Data were analyzed with Minitab software (version 18, USA), and the difference between the means was determined using Student’s *t*-test at *p* ≤ 0.05. 

## Supplementary Materials

The following are available online at https://www.mdpi.com/2223-7747/8/11/446/s1, Table S1: Primers used for real time RT-PCR assays.
