# Supplementary material for: Enhancement of Drought Tolerance in Cucumber Plants by Natural Carbon Materials
Source: Plants (Basel). 2019 Oct 24;8(11):446. doi: 10.3390/plants8110446 (PMC6918154; doi:10.3390/plants8110446)
Supplement: Supplementary file 1 [file plants-08-00446-s001.pdf]

Supplementary Table

Table S1. Primers used for real time RT-PCR assays.

Gene	Primer pairs
CsEF1α	F : 5'-GATGATTTGCTGCTGCAACAAGATG-3'<br>R : 5'-TTGTACCAGTCAAGGTTGGTCGACC-3'
CsPYL1	F : 5'-TTTGGAGATGGACAGGCAGGAG-3'<br>R : 5'-AAGCATACACCACCATGGACAAAAC-3'
CsPYL8	F : 5'-CATTCAAAGGCACCACAACC-3'<br>R : 5'-GCTCCTGACCAACGACCATA-3'
CsSnRK2.1	F : 5'-TCGCAACCTTCTTTCTCGC-3'<br>R : 5'-ATTTCCTCAACGCTCTGTG-3'
CsSnRK2.2	F : 5'-ATTTGCGACTTCGGCTATTC-3'<br>R : 5'-TAAGCTCCAACCAGCATCAC-3'
CsRD29B	F : 5'-AAGGTGAAGAGCGAAAC-3'<br>R : 5'-CAGGAGCAAGGCGTTTA-3'

F indicates forward and R indicates reverse.
